# Valuation of the EQ-5D-5L in Taiwan

**DOI:** 10.1371/journal.pone.0209344

**Published:** 2018-12-26

**Authors:** Hsiang-Wen Lin, Chia-Ing Li, Fang- Ju Lin, Jen-Yu Chang, Churn-Shiouh Gau, Nan Luo, A. Simon Pickard, Juan M. Ramos Goñi, Chao-Hsiun Tang, Chien-Ning Hsu

**Affiliations:** 1 School of Pharmacy and Graduate Institute, College of Pharmacy, China Medical University, Taichung, Taiwan; 2 Department of Pharmacy, China Medical University Hospital, Taichung, Taiwan; 3 Department of Pharmacy Systems, Outcomes & Policy, College of Pharmacy, University of Illinois at Chicago, Chicago, IL, United States of America; 4 Department of Medical Research, China Medical University Hospital, Taichung, Taiwan; 5 Graduate Institute of Clinical Pharmacy, College of Medicine, National Taiwan University, Taipei, Taiwan; 6 School of Pharmacy, College of Medicine, National Taiwan University, Taipei, Taiwan; 7 Department of Pharmacy, National Taiwan University Hospital, Taipei, Taiwan; 8 Center for Drug Evaluation, Taipei, Taiwan; 9 Saw Swee Hock School of Public Health, National University of Singapore, Singapore; 10 Department of Pharmacy System, Outcomes, and Policy, College of Pharmacy, University of Illinois at Chicago, Chicago, IL, United States of America; 11 Senior Scientist, EuroQol Research Foundation, Rotterdam, The Netherlands; 12 School of Health Care Administration, Taipei Medical University, Taipei, Taiwan; 13 Department of Pharmacy, Kaohsiung Chang Gung Memorial Hospital, Kaohsiung, Taiwan; 14 School of Pharmacy, Kaohsiung Medical University, Kaohsiung, Taiwan; Sciensano, BELGIUM

## Abstract

**Objectives:**

To date, a value set for the EQ-5D-5L based on the health state preferences of the general Taiwanese population has not been available. This study aimed to develop a Taiwanese value set for EQ-5D-5L to facilitate health technology assessment for medical products and services.

**Methods:**

An international standardized protocol for EQ-5D-5L valuation studies developed by the EuroQol group was adopted. Adult members of the general public were recruited from six geographic regions in Taiwan. In computer-based face-to-face interviews, each participant completed 10 composite time trade-off (C-TTO) tasks and 7 discrete choice experiment (DCE) tasks. The C-TTO and DCE data were modeled alone or in combination (using hybrid models) with additive models containing 20 dummy variables as main effects. The model performance was assessed both quantitatively and qualitatively (mainly logical consistency and prediction patterns).

**Results:**

Of 1,073 recruited participants, 1,000 completed the study. Approximately 13% of observed utility values were -1 in the C-TTO tasks. The hybrid model, using all available data that assumed C-TTO response values left-censored at -1 and with main effects coefficients with logical consistency (monotonicity), was considered as the most appropriate model. The predicted utility ranged from -1.0259 to 1.

**Conclusions:**

An EQ-5D-5L value set was developed for Taiwan using an established study protocol and a representative sample of the general population. This may facilitate health economic evaluations and decision making on resource allocation under Taiwan’s national health insurance program in the future.

## 1. Introduction

The National Health Insurance (NHI) program is compulsory for all citizens and employees in Taiwan. Given increasing financial pressures to implement and maintain the NHI in Taiwan [1], health technology assessment has been introduced to the Second Generation Taiwan NHI program to facilitate decision making related to new health technologies by Ministry of Health and Welfare [2]. Cost-effectiveness analysis of new medications, medical devices and interventions in the Center for Drug Evaluation is encouraged under the policy of National Health Insurance Administration in Taiwan [3]. The official guidelines for cost-effectiveness analysis and budget impact analysis, published by the Center for Drug Evaluation, have recommended using health utility measures in order to facilitate cost-effectiveness analysis [3], in which the EuroQol five-dimensional (EQ-5D) measure was listed as one of the recommended instruments.

The EQ-5D instrument is the most widely used generic preference-based measure of health. The questionnaire includes five dimensions related to health (mobility, self-care, usual activities, pain/discomfort, and anxiety/depression), originally with three levels each, as well as a self-rating of health using a visual analogue scale (EQ-VAS) [4]. The Taiwan version of the three-level EQ-5D (EQ-5D-3L) was introduced into the National Health Interview Survey in 2009 [5], but switched to a five-level format in 2017. The EQ-5D-3L is listed as one of five main outcome measures in the “Quality Enhancement Pilot Project”, sponsored by the National Health Insurance Administration [6]. The version of the EQ-5D that expands the number of levels to five (“EQ-5D-5L”) has been demonstrated to have enhanced discriminative ability [7]. To facilitate valuation of the EQ-5D-5L, a standardized international protocol was created [8], and a valuation task software was developed (*i*.*e*. the EQ-VT) to promote international harmonization and improve data quality [9]. Numerous countries have published EQ-5D-5Lvalue sets using the standardized protocol [10–17], with more forthcoming.

So far, there has not been an EQ-5D-5L value set based on the health state preferences of the general Taiwanese population. A previous study elicited values for EQ-5D-3L in Taiwan, but it was based on health preferences of a non-representative sample in the hospital setting [18]. To facilitate decision-making based on the values of the Taiwanese general population, a country-specific value set for a preference-based measure of health is desirable. The objective of this study was to develop a value set for the EQ-5D-5L based on the preferences of a representative sample of the general population in Taiwan.

## 2 Methods

This study used the EQ-5D-5L valuation protocol, interview guide for data collection, and a computerized interview program (*i*.*e*. EuroQol Valuation Technology version 2.0, EQ-VT 2.0) [8].

### 2.1 Participants

Members of the general population were recruited from nine randomly selected cities located in six geographic regions in Taiwan: Taipei (Taipei and New Taipei), north (Tauyuan and Chinju), east (Hualien), central (Taichung), south (Chiayi, Tainan), and KauPing (Kaohsiung). The nine cities are representative of the three main living areas (north & east, central, south). Multi-stage stratified quota sampling was used to ensure that participants from each region had similar characteristics in terms of age, gender, and educational level and, if possible, similar characteristics to the population they represented [19]. To assess the representativeness of the observed C-TTO values from the nationwide sample, a subsample that matched the general population based on age, gender, living area, and education was generated by random selection from the full sample. The observed C-TTO values of this subsample were compared with that obtained from the full sample.

Potential participants living in the nine cities were referred to the investigators by local leaders or persons-in-charge in community centers, owners of street shops, school teachers, pharmacists in community pharmacies, or participants themselves. All potential participants were contacted via postcards or referred by referees to determine their willingness to be interviewed in either their homes or in a safe, undisruptive public location. Study participants were aged 20 or older, able to understand the valuation tasks, able to read and communicate in Chinese or Taiwanese, and provided informed consent. Those participants who appeared to have any acute illness or cognitive problems, did not want to talk about death as a hypothetical scenario, or who rushed to complete the interviews based on the interviewer’s observation were excluded from the final data analysis. Participants who completed the interview received NT$ 200 for their time and participation.

A required sample size of 1,000 respondents was estimated based on obtaining a 0.01 standard error (SE) for observed mean C-TTO values in the hybrid model [20]. The study was approved by the China Medical University & Hospital Research Ethics Committee (CMUH105-REC1-111).

### 2.2 Instruments

#### 2.2.1 EQ-5D-5L health states

We used the official EQ-5D-5L traditional Chinese character Taiwan version. EQ-5D-5L describes 3,125 (5^5^) possible health states. Each health state described by the EQ-5D-5L classifier can be represented by a five-digit number, one for each of the five dimensions. For instance, “21111” refers to a state in which a person has slight problems with walking, but no problems in the remaining four dimensions, whereas “54123” represents a person unable to walk about, with severe problems washing or dressing, no problem performing usual activities, slight pain or discomfort, and moderate anxiety or depression [7]. Because the traditional Chinese characters used in the EQ-5D-5L to express the different severity levels (*i*.*e*. no problems, slight problems, moderate problems, severe problems, extreme problems) look very similar, each descriptor was highlighted in red for all levels of the health states shown to respondents by the EQ-VT program. This allowed respondents to differentiate the severity of the dimensions of a health state more easily.

#### 2.2.2 Valuation procedures

A face-to-face, computer-based interview was conducted with each participant. Two elicitation techniques, *i*.*e*. the composite TTO (C-TTO) and discrete choice experiment (DCE), were applied by following the standard procedures developed by the EuroQol Group [8]. The valuation interview included the following sections in their order of presentation: self-reported EQ-5D-5L descriptive system and EQ VAS, background questions (age, gender, experience of illness), one C-TTO exercise using being in a wheelchair as an example, three C-TTO practice exercises (the response data were not included in modeling), ten C-TTO tasks, a feedback module for C-TTO tasks, three debriefing questions on the C-TTO, seven DCE tasks, three debriefing questions on the DCE, and a general comments section at the end. After the ten C-TTO tasks, participants were presented with the rank ordering of health states based on their C-TTO observations and asked to identify heath state valuation(s) they considered inappropriately located in the ranking(s). This allowed the respondents to identify problematic valuation tasks in the feedback module for C-TTO tasks. In the C-TTO tasks, 86 health states were selected using Monte Carlos simulations over ten blocks with a similar level of severity [8]. Each block contained a very mild state (*e*.*g*. 21111), the worst state (*i*.*e*. 55555), and a balanced set of intermediate states [8]. The EQ-VT platform randomly assigned respondents to one of the ten C-TTO blocks and presented the health states in random order.

#### C-TTO

The C-TTO tasks were designed as a series of questions asking the respondent to compare between Life A, which varied the length of time in full health, and a Life B, which presented a scenario of less than full health as described by the EQ-5D-5L health states for a fixed length of time. Details regarding C-TTO can be found in a study by Janssen et al. [21]. Briefly, the C-TTO incorporated traditional TTO to elicit the value of health states considered to be better than death (BTD) and lead-time TTO for a health state worse than death (WTD) by adding additional healthy years (“lead time”) for trading WTD tasks.

In the C-TTO protocol, the respondent is first asked to choose either 10 years in an impaired health state (Life B) or 10 years of full health (Life A) and follow a series of choice-based questions (Life A or B) to identify indifferences between the length of time in Life A as that the period of time (“x”) in Life B. Thus, the Life B health state (*i*.*e*. BTD) is defined as x/10. For instance, if a respondent felt 5 years and 6 months of full health in Life A is about the same of 10 years living in a specific impaired health state of Life B, the value for this health state would be 0.55 (5.5/10). When respondents preferred immediate death to 10 years in the specified impaired health state, the leading-time C-TTO task was introduced to value the WTD health state (below 0). In this way, whenever the respondents preferred to go for “about the same” between a period of time (“x”*)* in Life A and in the impaired health of Life B, the value for Life B health state in WTD tasks would be (*x*-10)/10. The lowest value would be -1 of a given health state. Further, the percentages of observed C-TTO values, divided into 23 categories (i.e. -1, -0.99~-0.9, -0.89~-0.8, …0…0.80~0.89, 0.90~0.99, 1) for the full sample, sample without flagged QC and sample with matched education, were compared using the chi-squared test.

#### DCE

DCE is a choice-based technique that involves pairwise comparisons [22], and is included in the EQ-VT protocol as DCE without duration such that it generates health preferences on a latent scale [8]. Compared to C-TTO tasks, DCE tasks tend to be considered easier to comprehend and less time-consuming for the participants. The DCE design consisted of 196 pairs of EQ-5D-5L health states distributed over 28 blocks [8]. Each respondent was assigned to a block that included seven pairs of health states, presented in random order by the EQ-VT platform.

### 2.3 Quality assurance procedures

Interviewer training and quality assurance of data collection process have been shown to be critical to achieve high-quality preference data [17]. Potential interviewers underwent the standardized training programs developed by the research team. Only those interviewers who demonstrated satisfactory performance were chosen for the formal valuation study. A pilot study with 50 participants was performed to test the data collection procedures and evaluate the traditional Chinese version of the EQ-VT program. In the formal valuation study, the research team worked together with a supporting team from the EuroQol Group to monitor interviewers’ behaviors and provide cyclic feedback and retraining, if necessary, to ensure good quality interviews. The interview feedback took the form of weekly quality assurance reports that were disseminated to an individual interviewer using instant communication freeware (*i*.*e*. LINE) to facilitate smooth and instant communications between interviewers and the research team.

Quality assurance was assessed in terms of how interviewers explained the C-TTO example and time spent on the formal 10 C-TTO tasks. An interview would be flagged for investigation if any of the following four quality controls (QCs) was observed: (1) time spent on explaining the wheelchair example < 3 minutes; (2) no explanation of the lead-time TTO in the wheelchair example; (3) total time spent on the formal 10 C-TTO tasks < 5 minutes; and (4) significant logical inconsistency in the derived C-TTO values [9]. Unusual response patterns to DCE tasks of all A, all B, or alternative of AB (i.e. ABABABA or BABABAB) would also be flagged as well.

The EQ-VT program, interviewer training materials, interviewer guide, and other relevant materials (*e*.*g*., script for introducing this research to participants) were all translated from English into traditional Chinese characters using an iterative translation procedure.

### 2.4 Statistical analysis

All data were examined based on the quality criteria set by the EuroQol Group. Participants with missing responses to C-TTO and DCE tasks were excluded. The full sample and the subgroup sample without flagged QCs (after excluding 30 participants due to quality concerns) were compared for their basic demographic characteristics, self-reported EQ-5D-5L outcomes, and observed C-TTO values. Because there is no recommended modelling protocol provided by the EuroQol Group for the EQ-5D-5L valuation data, the modelling approaches vary across countries to reveal their own data characteristics. While the results obtained from C-TTO and DCE models can be seen as complementary preference information to produce a value set individually, the results also can be combined to estimate utility values in order to maximize the product of likelihood functions in the hybrid model [23–24]. In this study, we assessed the C-TTO models and the DCE models independently and adapted the hybrid model [23–26]. All statistical analyses were performed using SAS 9.4 (SAS Institute, Cary, NC, USA), except the hybrid models, which were estimated using STATA 14 (Stata Corp LP, College Station, TX, USA) using the hyreg command [24].

#### 2.4.1 Model construction

The goal was to use all the observed preferences and obtained data to predict utility function for all health states in this study. The 20 dummy variables without interactions were included in each model to estimate the main effect as that addressed in previous studies [23–26]. Each dummy variable represented the additional utility decrement of moving from one level to the next worse level of the same dimension (*i*.*e*. incremental-dummies). For instance, the disutility of level 5 in the MO dimension was represented by the sum of disutility of MO level 2, MO level 3, MO level 4 and MO level 5. The total disutility of a health state could then be calculated as the sum of disutility for all five dimensions (5x4 = 20 dummies).

Four estimation methods were used for the C-TTO models, including ordinary least square (OLS), general least square (GLS), Tobit and Tobit-GLS regression. Tobit regressions were used to estimate the relationships between variables in the C-TTO observations censored at -1. The DCE data were assessed by the conditional logistic regression models that evaluate the distance of preferences between two specific health states. Since the DCE pairs contained health states presented with no specified duration, DCE responses were modeled initially on an arbitrary latent scale and required rescaling to be interpretable on the desired utility scale [22, 26]. The two hybrid methods, *i*.*e*. the standard hybrid model and the hybrid-Tobit model (censored at -1), were used to maximize the single likelihood of continuous or dichotomous responses in this study [23–24]. In particular, the rescaled DCE coefficient equals the original DCE coefficient divided by theta derived from the corresponding hybrid model [22] and the hybrid models were processed with the STATA hyreg command.

#### 2.4.2 Model performance

The criteria used to select the most appropriate model included (a) mainly logical consistency (monotonicity), that is, worse health states should have lower values than better health states, as the primary criteria (b) goodness of fit, and then (c) distribution of predicted utility. The Akaike information criterion (AIC) and Bayesian information criterion (BIC) were calculated to evaluate the goodness-of-fit of either the C-TTO or DCE models, but not the appropriate criteria for hybrid models. Log-likelihood was used to present the differences between the hybrid model and both C-TTO and DCE models separately. The final models with each of the C-TTO, DCE and of hybrid approaches were evaluated by estimated utilities of the 86 health states and model performance. The inclusion of constant was tested in the final models. If the variance of C-TTO data were not homogeneous among health states, the regression modeling considering heteroscedasticity or not was performed as well.

## 3 Results

### 3.1 Participant characteristics

Ten interviewers performed valuation interviews in the randomly selected nine cities in Taiwan from January 2017 to July 2017. A total of 1,123 participants received the valuation interviews. Among them, 50 participated in the pilot testing, and 73 discontinued the interviews in the C-TTO tasks section or rushed to complete the interview in the formal valuation study. These participants were excluded from the final data analysis. All basic characteristics of the full sample (N = 1,000) and the sample after excluding 30 participants whose interviews with quality concerns (*e*.*g*., due to insufficient time spent on the wheelchair C-TTO example or the 10 formal C-TTO tasks) (N = 970) were both comparable with the Taiwan adult general population [20], except the sample had 30% more participants with higher education and 30% less with primary school education (Table 1). In that regard, a subsample (n = 295) matched by age, gender, living area, and education to the full sample were identified and their observed TTO values with those of the full sample were analyzed and compared afterward.

**Table 1:** Characteristics of respondents compared to Taiwan adult general population.

Characteristics	Full sample	Subgroup sample without flagged QC reports	Taiwan adult general population*	Differences^#^ (%)
Sample size	1000	970	18,963,159	NA
Gender				
Female	505(50.5%)	489(50.4%)	9,627,275(50.8%)	-0.30%
Male	495(49.5%)	481(49.6%)	9,335,884(49.2%)	0.30%
Age				
20~29	173(17.3%)	167(17.2%)	3,375,442(17.8%)	-0.50%
30~39	208(20.8%)	200(20.7%)	4,058,116(21.4%)	-0.60%
40~49	190(19.0%)	187(19.3%)	3,621,963(19.1%)	-0.10%
50~59	193(19.3%)	187(19.3%)	3,603,001(19.0%)	0.30%
>60	236(23.6%)	229(23.6%)	4,304,637(22.7%)	-0.90%
Living area				
North &East	500(50.0%)	484(49.9%)	9,614,322(50.7%)	-0.70%
Central	225(22.5%)	218(22.5%)	4,077,079(21.5%)	1.00%
South	275(27.5%)	268(27.6%)	5,271,758(27.8%)	-0.30%
Education				
Primary sch.	130(13%)	125(12.9%)	8,362,753(44.1%)	-31.10%
High sch.	277(27.7%)	268(27.6%)	5,461,390(28.8%)	-1.10%
Higher education	593(59.3%)	577(59.5%)	5,139,016(27.1%)	32.20%
Employment				
Full Paid	694(69.4%)	676(69.7%)	NA	NA
Part-time	97(9.7%)	95(9.8%)	NA	NA
Unemployed	209(20.9%)	199(20.5%)	NA	NA

*Taiwan general adult population = data from 2016 for all population with aged 20 or more

#Difference = Proportion in full sample- Proportion in Taiwan adult general population

### 3.2 Data characteristics

**Fig 1:**
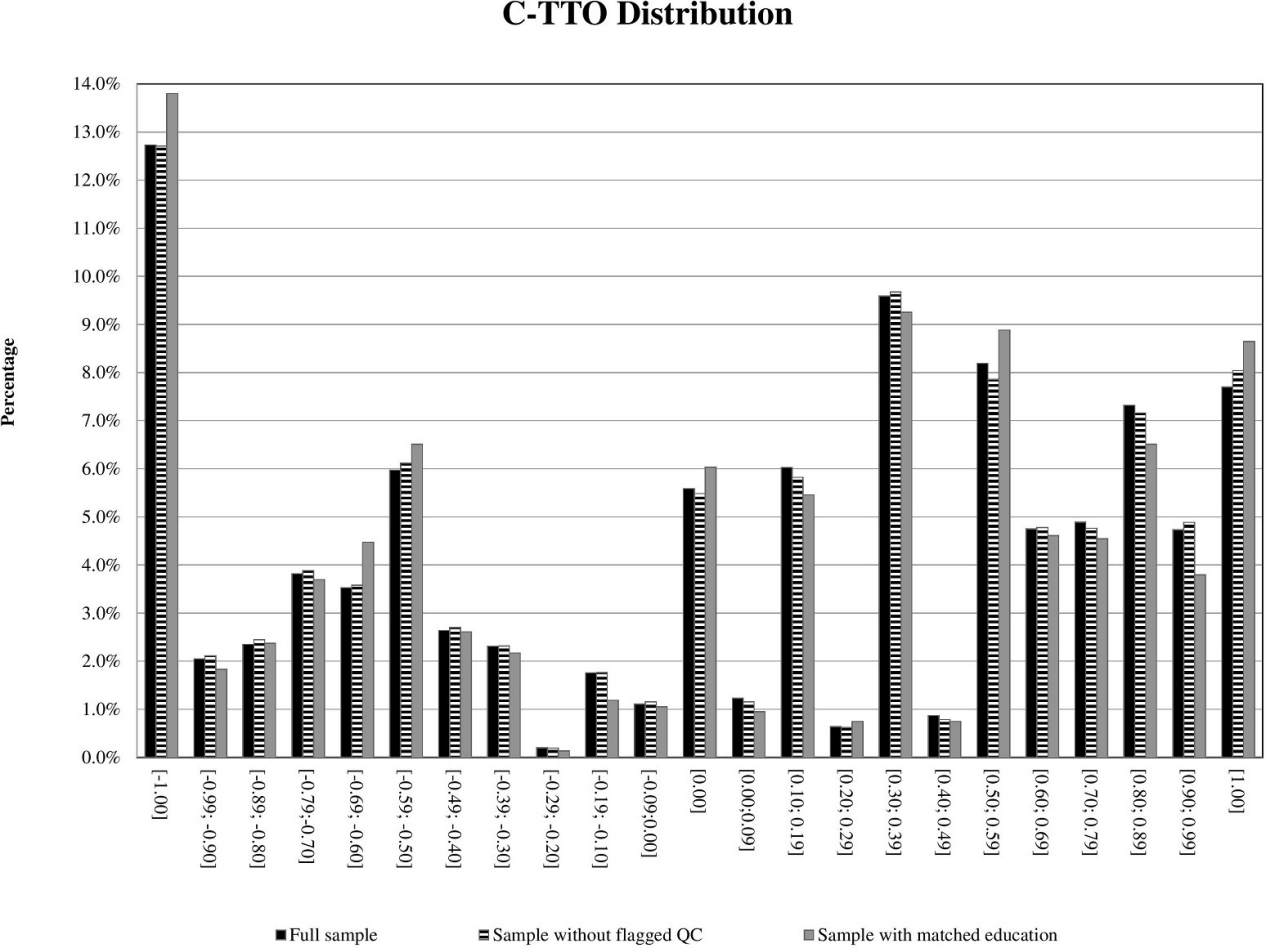
Distribution of C-TTO values.

The data obtained from all the 1,000 participants, including those 30 participants with flagged responses on the C-TTO tasks, were kept in the final modeling analysis. While the C-TTO dataset comprised 10,000 observations, the DCE dataset comprised 7,000 observations, including observations from unusual response patterns provided by 21 respondents (2.1%). The distribution of observed C-TTO values in the education-matched subgroup (n = 295) was comparable with that of the full sample (N = 1,000) and the subgroup of participants without quality concerns (N = 970) (Fig 1). The percentages of C-TTO responses, divided into 23 categories, were not significantly different among these three samples (*p* = 0.384). Therefore, responses obtained from all 1,000 respondents were used for the following modeling. In the full sample, more than half of the C-TTO values were positive (55.9%), whereas 7.7% were “1”, 5.6% were “0”, 38.5% were negative, and 12.7% were “-1”.

### 3.3 Modelling results

The estimation results for C-TTO models are presented in Table A in S1 File. There were 12.7% (1,273) left-censored C-TTO observations. The lowest possible value was -0.9583 in the Tobit GLS model censored at -1 and 0.9664 in the Tobit model censored at -1, respectively. These two lowest values seemed more appropriate given the spike on the left tail of the observed C-TTO distribution than that obtained from OLS model and GLS model. Of these models, the Tobit GLS model censored at -1 had relatively lower AIC and BIC values than the OLS and Tobit models censored at -1, but not for GLS model.

The lowest possible values for the predicted utility derived from the DCE model without the intercept constant and rescaled using theta derived from hybrid models was -1.0065 (Table B in S1 File). Importantly, in the DCE models, the coefficients for the main effects were almost the same with or without intercept constants before rescaling (Tables B and C in S1 File). Furthermore, the lowest possible values for the predicted utility in the hybrid models with C-TTO values censored at -1 and also considering heteroscedasticity or not were -1.2362 and -1.0259, respectively (Table D in S1 File). These values were all lower than the value obtained from the standard hybrid model (*i*.*e*. -0.8602). The intercept constant appeared to be not significant (P value: 0.228) in the Tobit model and in the logistic model (P value: 0.927), so we excluded all the intercept constants.

The estimation results of the best fit model with each of the C-TTO, DCE, and hybrid approaches are presented in Table 2 and Table 3. All the main effects coefficients in the models showed logical consistency (monotonicity), *i*.*e*. the coefficients, which represent the additional utility decrement of moving from one level to another, were positive. Although there was no uniform pattern, the greatest decrease in utility values in a dimension appeared to take place in moving from moderate to severe problems or sometimes from slight to moderate problems. The results of the hybrid models (Table 2), which utilized the C-TTO and DCE data, were in good agreement with both C-TTO and DCE models in each dimension. The estimated utility values (Table 3) for the commonly selected health states show that DCE-based scores tended to be higher than C-TTO-based values for all states, except for some poorer health states.

**Table 2:** Estimation results for C-TTO, DCE, and hybrid models (for full sample).

	C-TTO	DCE	Hybrid
Independent variables	Tobit GLS model censored at -1	Conditional logistic model, rescaled using theta derived from hybrid-Tobit model censored at -1	With C-TTO values censored at -1 without considering heteroscedasticity and constant
Mobility (MO)			
No to slight problem	0.1054 (0.0132)^¶^	0.0796 (0.0162)^¶^	0.1076 (0.0112)^¶^
Slight to moderate problems	0.1204 (0.0148)^¶^	0.0939 (0.0170)^¶^	0.0920 (0.0124)^¶^
Moderate to severe problems	0.1336 (0.0163)^¶^	0.1631 (0.0158)^¶^	0.1656 (0.0125)^¶^
Severe to extreme problems	0.0967 (0.0159)^¶^	0.1517 (0.0168)^¶^	0.1115 (0.0122)^¶^
Self-care (SC)			
No to slight problem	0.0816 (0.0127)^¶^	0.0111 (0.0176)	0.0757 (0.0110)^¶^
Slight to moderate problems	0.0947 (0.0158)^¶^	0.0557 (0.0175)^¶^	0.0565 (0.0127)^¶^
Moderate to severe problems	0.0714 (0.0161)^¶^	0.1604 (0.0174)^¶^	0.1322 (0.0129)^¶^
Severe to extreme problems	0.0718 (0.0142)^¶^	0.0749 (0.0162)^¶^	0.0597 (0.0117)^¶^
Usual Activities (UA)			
No to slight problem	0.0569 (0.0133)^¶^	0.0489 (0.0161)^¶^	0.0726 (0.0111)^¶^
Slight to moderate problems	0.0883 (0.0151)^¶^	0.0117 (0.0163)	0.0508 (0.0119)^¶^
Moderate to severe problems	0.1504 (0.0163)^¶^	0.1794 (0.0165)^¶^	0.1568 (0.0124)^¶^
Severe to extreme problems	0.0283 (0.0158)	0.1005 (0.0170)^¶^	0.0703 (0.0124)^¶^
Pain/Discomfort (PD)			
No to slight problem	0.0790 (0.0118)^¶^	0.0764 (0.0167)^¶^	0.0868 (0.0108)^¶^
Slight to moderate problems	0.1006 (0.0164)^¶^	0.0342 (0.0166)	0.0710 (0.0124)^¶^
Moderate to severe problems	0.1636 (0.0155)^¶^	0.2038 (0.0167)^¶^	0.1824 (0.0124)^¶^
Severe to extreme problems	0.0901 (0.0169)^¶^	0.1681 (0.0175)^¶^	0.1132 (0.0127)^¶^
Anxiety/depression (AD)			
No to slight problem	0.0579 (0.0134)^¶^	0.0322 (0.0173)	0.0637 (0.0113)^¶^
Slight to moderate problems	0.1480 (0.0157)^¶^	0.1143 (0.0166)^¶^	0.1192 (0.0125)^¶^
Moderate to severe problems	0.1406 (0.0150)^¶^	0.1453 (0.0176)^¶^	0.1572 (0.0124)^¶^
Severe to extreme problems	0.0790 (0.0140)^¶^	0.1013 (0.0170)^¶^	0.0811 (0.0119)^¶^
Range of possible values	[-0.9583, 1]	[-1.0065, 1]	[-1.0259, 1]
Log likelihood	-6180.705	-3076.2541	-11197.917
AIC	12405.410	6192.5083	22439.834
BIC	12564.038	6328.5166	22609.660
RMSE	0.4729	NA	NA
MAE	0.3601	NA	NA

Model estimates are presented as coefficient (SE).

^¶^*p* value <0.01.

AIC, Akaike information criteria; BIC, Bayesian information criteria; GLS, generalized least squares; MAE, mean absolute error; OLS, ordinary least squares; RMSE, root mean square error.

**Table 3:** Selected predicted utility in C-TTO, DCE, and hybrid models (for full sample).

	C-TTO	DCE	Hybrid
Independent variables	Tobit GLS model censored at -1	Conditional logistic model, rescaled using theta derived from hybrid-Tobit model censored at -1#	With C-TTO values censored at -1 without considering heteroscedasticity and constant
Estimated utility values			
U(21111)	0.8946	0.9204	0.8924
U(12111)	0.9184	0.9889	0.9243
U(11211)	0.9431	0.9511	0.9274
U(11121)	0.9210	0.9236	0.9132
U(11112)	0.9421	0.9678	0.9363
U(12345)	0.0045	0.2208	0.0395
U(42114)	0.2125	0.3605	0.2190
U(33511)	0.2740	0.4192	0.3177
U(25331)	0.2503	0.4471	0.2871
U(35411)	0.1591	0.2844	0.1961
U(34511)	0.2026	0.2588	0.1855
U(35412)	0.1012	0.2522	0.1324
U(33531)	0.0944	0.3086	0.1599
U(55512)	-0.1574	-0.1631	-0.2150
U(52533)	-0.2471	-0.0970	-0.2436
U(34544)	-0.4871	-0.3474	-0.4948
U(34553)	-0.4366	-0.3702	-0.4508
U(55433)	-0.4567	-0.2875	-0.4217
U(35552)	-0.3604	-0.3308	-0.3913
U(54454)	-0.7792	-0.7298	-0.8148
U(55444)	-0.7609	-0.6366	-0.7613
U(55552)	-0.5907	-0.6456	-0.6684
U(54455)	-0.8582	-0.8311	-0.8959
U(55554)	-0.8793	-0.9052	-0.9448
U(55545)	-0.8682	-0.8384	-0.9127

Model estimates are presented as coefficient.

**Fig 2:**
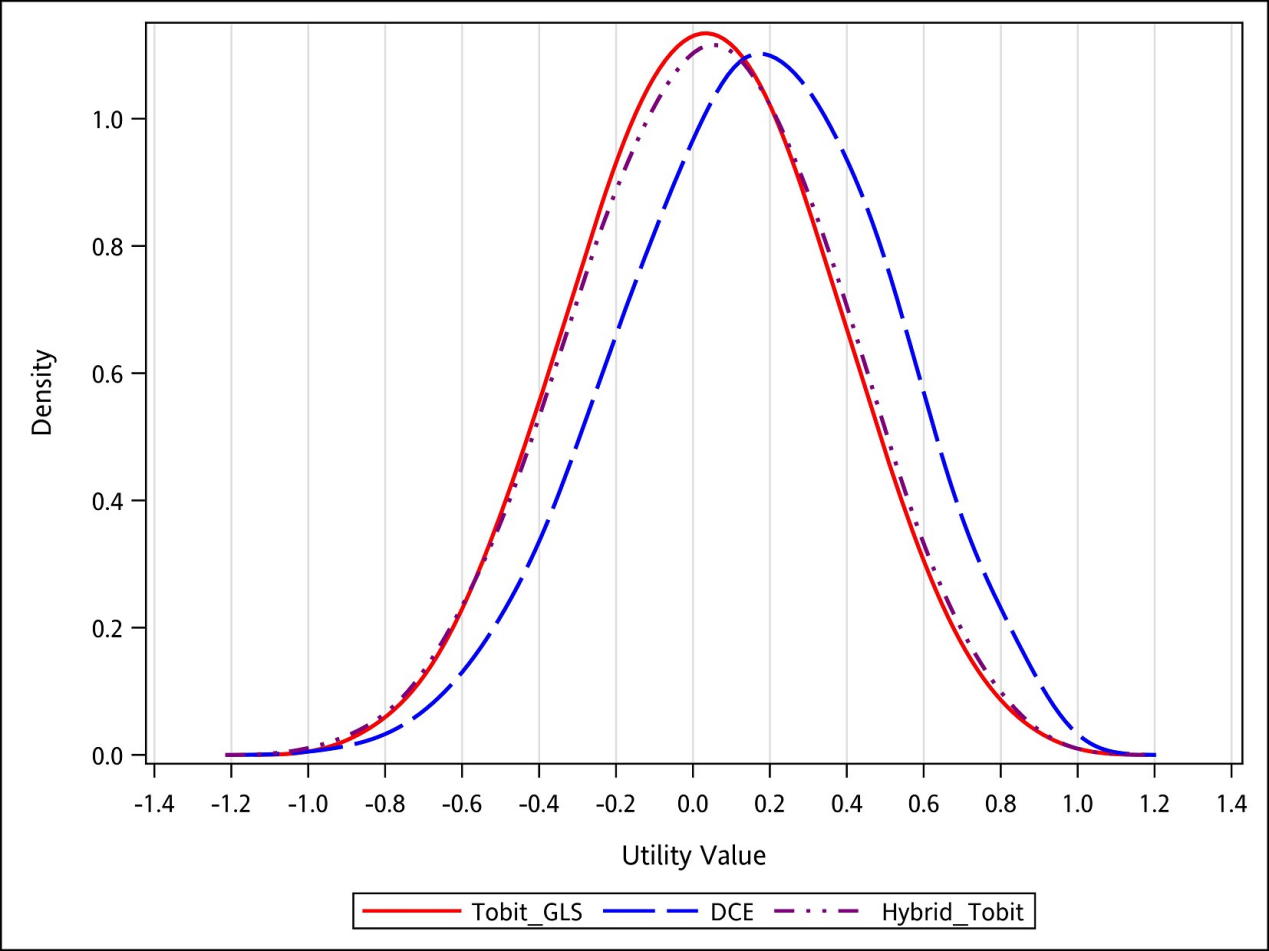
Kernel density function of C-TTO, DCE and hybrid predicted utilities; total in 3125 health states.

The predicted values for the sorted 86 health states showed that the hybrid model censored at -1 was similar to the Tobit GLS model censored at -1 and the observed C-TTO, in relative agreement with the DCE model (S1 Fig). The kernel density functions of predicted values for 3,125 health states also provided similar patterns as for the aforementioned 86 health states (Fig 2). Considering all these quantitative and qualitative findings and using all the observed data, the hybrid model assuming left-censoring of the C-TTO at -1 excluding heteroscedasticity and constant was identified as the most preferred model. Using this preferred model to value EQ-5D-5L health states, the maximum tariff was 1 for full health (health state “11111”) followed by the health state “11112” with a value of 0.9363 (Table 3). To obtain utility for an EQ-5D-5L health state, for instance “12345”, using the final preferred value set, we established the following calculation: Utility weight (“12345”) = 1 –(0)–(0.0757)–[(0.0726)+(0.0508)]–[(0.0868)+(0.0710)+(0.1824)]–[(0.0637)+(0.1192)+(0.1572)+(0.0811)] = 0.0395.

## 4 Discussion

Using both C-TTO and DCE data derived from a sample representative of the Taiwanese adult general population, we identified the most appropriate value set of EQ-5D-5L for Taiwan. The basic demographic characteristics of the respondents were representative with respect to sex, age group, and living area, but they were generally more educated than the general Taiwan population. According to the subgroup analysis, the C-TTO observed valuation patterns do not appear to be affected by the level of education. Thus, this makes the derived EQ-5D-5L value set suitable for use in economic evaluations of healthcare interventions in Taiwan.

A large proportion of C-TTO observations considered WTD (negative values) were observed in Taiwan (38.5%), Hong Kong, and Indonesia (36%) [15, 16], which was not observed in the value sets in other Asian countries (*e*.*g*. 10% in China, 0.1% in Japan and South Korea [12, 27, 28]) and western countries (2% in the Netherlands, 4.55% with -1 in Spain) [13, 17]. While Taiwan has a comprehensive NHI program, which differs than those health care systems in Indonesia and Hong Kong, it is possible that Taiwanese people might have adapted to some mild level of health problems but tend to give up their lives to avoid living in many of the poorer health states. Furthermore, it is unknown whether cultural variations could contribute to these differences or not. Taking China as an example, there were different observed and predicted C-TTO distributions in our study compared to those in China, even if the cultural similarity is high in some sense. The different demographic characteristics, including age, education, and employment status between respondents might be influential in such differences [29]. While the Chinese study was conducted in five selected large urban centers, our study was performed in nine randomly selected cities located in six geographical regions (to reflect the characteristics of the general population in Taiwan). More research is needed to explore the similarities and/or differences of observed and predicated C-TTO distributions in Taiwan, China, Hong Kong and other countries in Asia, as well as in Europe or America.

While the sum of coefficients on the mobility dimension was the highest and was relatively higher than the other dimensions in Indonesia and Hong Kong, which used hybrid models as well, the sum of coefficients for pain/discomfort and anxiety/depression were very close to those for mobility in Taiwan. Disutility values using the hybrid model for mobility, pain/discomfort, and anxiety/depression were 0.4767, 0.4534, 0.4212, respectively, in our study. This implies that, unlike the derived value sets in Indonesia and Hong Kong, where the mobility dimension affected utility the most, problems with pain/discomfort and anxiety/depression were as influential on utility estimation as that of mobility in Taiwanese people. In other words, these findings may suggest that people in Indonesia and Hong Kong might consider a deficiency in their mobility relatively more than that on anxiety/depression and/or pain/discomfort dimensions, but this is not the case for the Taiwanese population. Further studies to explore the reasons beyond having a different impact on dimensions (*i*.*e*. the specific dimension with the most/less/similar influential on utility or health-related quality of life) of the Taiwanese and other populations is needed.

The predicted utility score to the selected health state 21111 in our study was similar to those in the models from Indonesia and Hong Kong (0.881, 0.8879, and 0.8924, respectively). In contrast, the lowest utility scores for the worst health state 55555 in Indonesia and Hong Kong were -0.865, and -0.8637, respectively, but this was -1.0259 in Taiwan. Thus, more research is needed to explore the causes of these differences across populations, such as understanding the social-cultural value of the worst health states.

We used the hybrid model combining C-TTO and DCE data to develop the value set for EQ-5D-5L [23] and compared the results to earlier approaches to optimize the use of information closer to the nature of the data [10]. In our results, the coefficients derived from the conditional logistic regression model were on the latent arbitrary utility scale. Thus, they were rescaled using the same parameter obtained from the hybrid model, as has been done in other studies [23, 30]. The coefficients from the hybrid model with 20 main effects were consistent with the C-TTO and DCE models regarding pain/discomfort as the most important dimension and mobility and self-care as the least important. The main effects hybrid model produced a wider range of predicted utility values (between -1.0259 and 1) at the lower end of the scale compared to the other two methods. The lowest predicted utility with more than -1 could be because the coefficients were derived from the hybrid model (*i*.*e*. designated with four digits) and/or there was a greater impact of the DCE data in the hybrid model.

The more negative utility values might influence the future calculation of quality-adjusted life years (QALY) in cost-utility analyses using the Taiwan value set, as in the other studies [31, 32]. When adopting a national set of EQ-5D-5L weights for QALY calculations, decision-makers should be aware of obtaining a wider mean predicted utility based on a country-specific value set. This finding will affect the quality of life from 0~1 to -1.0259 ~1 for the QALY estimation accordingly. Compared to the current study, in fact, the derived value set in Lee’s EQ-5D-3L valuation study was less representative, because this study was conducted based on 745 respondents who were either employees or volunteers in 17 hospitals in Taiwan [18]. Only responses from 456 participants (61.2% of all participants) were used for value set modeling. In particular, more than 50% of the health states were considered worse than death responses in TTO tasks, and 23 participants gave negative values for all health states and were thus excluded from the final generalized estimating equation modeling. In other words, the participants’ characteristics and reported TTO values in Lee’s study were far different from those in the current study. The estimated value for the worst health state (*i*.*e*. 33333) based on the derived final N3 model (level 3 occurred within at least one dimension) in Lee’s study was 1.158, which was even lower than the lowest value in this EQ-5D-5L valuation study. Thus, further studies to explore the reasons for these findings are necessary.

It is necessary to consider some limitations of this study. Mainly, the distribution of basic demographic characteristics in the full sample is different on the education levels from the data reported by the Department of Household Registration, Ministry of the Interior. It was reported that 39% of Taiwanese held a higher education degree in 2012 [33], which was still lower than the proportion in this study (*i*.*e*. approximately 60%). With the extension of basic education from 9 to 12 years as compulsory education in Taiwan since 1983, people with primary education levels are usually above the age of 60 years and are mainly located in relatively rural areas, and not in the randomly sampled cities. Indeed, we encountered difficulty in recruiting participants with a primary school education level, and they seemed to have difficulty understanding health valuation tasks. Although education deviation seems to have a non-significant effect on the health state values in the present study, this highlights the importance of conducting a more representative sampling approach for a population-based value set in a country in order to better understand more representative social values that are not reflected in a previously drawn sample, such as socioeconomic situations and an aging population.

Unlike English and other languages, the traditional Chinese characters are more complicated and look very similar, especially for the different levels of severity. This situation might make the participants spend more time reading and differentiating the different levels of severity based on comments provided in the pilot study. Given more respondents tended to give up all times in the WTD tasks in our pilot study, our research team proposed that the EuroQol supportive group should make changes to the color coding of severity levels in order to facilitate formal valuation studies. Given that this is a big different change in valuation studies compared to those performed in other countries, it is unknown whether the highlighted levels of severity would stimulate the respondents to give up all life-year up to the maximum of 20 years. Further exploration about the impact of such changes is necessary.

We observed interviewer effects in Taiwan, which may be attributed to protocol violations, the quality of data, and EQ-VT platform data collection. Further studies should investigate which aspects of variability impose the greatest effect on modeling results. Moreover, in 2017, the five-level EQ-5D was successfully incorporated into the National Health Interview Survey study in Taiwan. This will facilitate more relevant data collection to explore the impact of clinical and sociodemographic variables on EQ-5D-5L dimensions and corresponding utility values derived from country-specific utility sets. Further research to explore the differences in country-specific utility sets and to investigate the real cause of the divergence of national value sets are needed in the future.

## 5. Conclusions

This paper reports the first EQ-5D-5L value set for Taiwan. The study design and data collection followed the EuroQol international valuation protocol and the preferred model reflects health preferences in the Taiwanese general population. Health preference weights have become increasingly important in economic evaluations of healthcare interventions. This Taiwan EQ-5D-5L value set is recommended for use in cost-utility analysis, health technology assessments for new drug and medical devices, and for the list formulary of National Health Insurance Administration and insurance coverage in Taiwan. This Taiwanese value set is expected to contribute to the evidence on the valuation of experienced health from a population perspective for decision makers concerning resource allocation decisions.

## Supporting information

S1 File Tables A-D.
(DOCX)

S1 Fig Comparing predicted values with observed C-TTO values; total in 86 health states. (TIF)

## Abbreviations

AIC: Akaike information criterion
BIC: Bayesian information criterion
BTD: Better than death
C-TTO: Time trade-off
DCE: Discrete choice experiment
EQ-5D-5L: EuroQol five-dimensional five-level
GLS: General least square
HTA: Health technology assessment
MAE: Mean absolute error
NHI: National Health Insurance
OLS: Ordinary least square
QCs: Quality concerns
RMSE: Root mean square error
SE: Standard error
VAS: Visual analogue scale
WTD: Worse than death
