# Correction: Valuation of the EQ-5D-5L in Taiwan

**DOI:** 10.1371/journal.pone.0305983

**Published:** 2024-06-18

**Authors:** Hsiang-Wen Lin, Chia-Ing Li, Fang- Ju Lin, Jen-Yu Chang, Churn-Shiouh Gau, Nan Luo, A. Simon Pickard, Juan M. Ramos Goñi, Chao-Hsiun Tang, Chien-Ning Hsu

There are errors in the Funding statement. The correct Funding statement is as follows: This study was sponsored by the EuroQol Research Foundation (EQ project 2016440), Center for Drug Evaluation (#10542652), the Ministry of Science and Technology, Taiwan (NSC102-2320-B-039-007) and the National Health Research Institute, Taiwan (Grant NHRI-EX103-10318PC, NHRI-EX104-10318PC, NHRI-EX105-10318PC, and NHRI-EX106-10318PC) in the course of the preparation and implementation stage of this project. These sponsoring agencies had no role in the study design, data collection and analysis, decision to publish or preparation of the manuscript.

There are errors in the Competing Interests statement. The correct Competing Interests statement is as follows: Although the listed 5th author (Dr. Churn-Shiouh Gau) and 7th author (Dr. A. Simon Pickard) acted as executive directors of the two major sponsoring agencies of this project (i.e., Center for Drug Evaluation, Taiwan, and EuroQol Research Foundation, respectively) when this study was carried out, these two and other co-authors declared no competing interests with these two sponsoring agencies, nor with the publication in PLOS ONE. This does not alter our adherence to PLOS ONE policies on sharing data and materials.

The Acknowledgement section for this paper is missing. The Acknowledgement section should be: The preliminary results of this study have been presented as a poster at 2nd EuroQol Academy Meeting 2017 at Grand Hotel in Noordwijk, the Netherlands, during May 6–8, 2016 and ISPOR 22nd Annual International Meeting in Boston during May 20–24, 2017. The authors would also like to express their gratitude to the study participants, research assistants, and interviewers for their contribution of time, specifically including Hsiang-En Chin, Jou-Ying Chen, Yueh-Lung Peng, Chih-Ting Lai and staff in Reach Data Technology (RDT) cooperation and those who involved in this study for their study assistance.
