# Supplementary material for: Valuation of the EQ-5D-5L in Taiwan
Source: PLoS One. 2018 Dec 26;13(12):e0209344. doi: 10.1371/journal.pone.0209344 (PMC6306233; doi:10.1371/journal.pone.0209344)
Supplement: S1 File — Tables A-D. (DOCX) [file pone.0209344.s001.docx]

**Table A: Estimation results for C-TTO models (for all samples)**

| Independent variables of the model |  | For all samples | | | | |  |
| --- | --- | --- | --- | --- | --- | --- | --- |
|  |  | OLS model | GLS model | Tobit model censored at -1 | Tobit GLS model censored at -1 |  |  |
| Mobility (MO) |  |  |  |  |  |  |  |
| No problems to slight problem |  | 0.1091 (0.0154)^¶^ | 0.1162 (0.0120)^¶^ | 0.0981 (0.0172)^¶^ | 0.1054 (0.0132)^¶^ |  |  |
| Slight problems to moderate problems |  | 0.1386 (0.0176)^¶^ | 0.1280 (0.0134)^¶^ | 0.1313 (0.0197)^¶^ | 0.1204 (0.0148)^¶^ |  |  |
| Moderate problems to severe problems |  | 0.1183 (0.0191)^¶^ | 0.1223 (0.0147)^¶^ | 0.1288 (0.0214)^¶^ | 0.1336 (0.0163)^¶^ |  |  |
| Severe problems to extreme problems |  | 0.0625 (0.0182)^¶^ | 0.0648 (0.0142)^¶^ | 0.0936 (0.0206)^¶^ | 0.0967 (0.0159)^¶^ |  |  |
| Self-care (SC) |  |  |  |  |  |  |  |
| No problems to slight problem |  | 0.0980 (0.0141)^¶^ | 0.0865 (0.0115)^¶^ | 0.0945 (0.0158)^¶^ | 0.0816 (0.0127)^¶^ |  |  |
| Slight problems to moderate problems |  | 0.0859 (0.0185)^¶^ | 0.0900 (0.0143)^¶^ | 0.0916 (0.0208)^¶^ | 0.0947 (0.0158)^¶^ |  |  |
| Moderate problems to severe problems |  | 0.0758 (0.0184)^¶^ | 0.0686 (0.0145)^¶^ | 0.0774 (0.0207)^¶^ | 0.0714 (0.0161)^¶^ |  |  |
| Severe problems to extreme problems |  | 0.0193 (0.0158) | 0.0281 (0.0126) | 0.0635 (0.0179)^¶^ | 0.0718 (0.0142)^¶^ |  |  |
| Usual Activities (UA) |  |  |  |  |  |  |  |
| No problems to slight problem |  | 0.0625 (0.0151)^¶^ | 0.0631 (0.0120)^¶^ | 0.0561 (0.0169)^¶^ | 0.0569 (0.0133)^¶^ |  |  |
| Slight problems to moderate problems |  | 0.0802 (0.0170)^¶^ | 0.0857 (0.0136)^¶^ | 0.0861 (0.0191)^¶^ | 0.0883 (0.0151)^¶^ |  |  |
| Moderate problems to severe problems |  | 0.1507 (0.0186)^¶^ | 0.1413 (0.0147)^¶^ | 0.1559 (0.0209)^¶^ | 0.1504 (0.0163)^¶^ |  |  |
| Severe problems to extreme problems |  | -0.0023 (0.0181) | -0.0075 (0.0141) | 0.0330 (0.0204) | 0.0283 (0.0158) |  |  |
| Pain/Discomfort (PD) |  |  |  |  |  |  |  |
| No problems to slight problem |  | 0.0912 (0.0134)^¶^ | 0.0868 (0.0107)^¶^ | 0.0832 (0.0149)^¶^ | 0.0790 (0.0118) ^¶^ |  |  |
| Slight problems to moderate problems |  | 0.0987 (0.0190)^¶^ | 0.0915 (0.0149)^¶^ | 0.1069 (0.0213)^¶^ | 0.1006 (0.0164)^¶^ |  |  |
| Moderate problems to severe problems |  | 0.1409 (0.0179)^¶^ | 0.1422 (0.0139)^¶^ | 0.1617 (0.0201)^¶^ | 0.1636 (0.0155)^¶^ |  |  |
| Severe problems to extreme problems |  | 0.0651 (0.0189)^¶^ | 0.0770 (0.0152)^¶^ | 0.0801 (0.0213)^¶^ | 0.0901 (0.0169)^¶^ |  |  |
| Anxiety/depression (AD) |  |  |  |  |  |  |  |
| No problems to slight problem |  | 0.0553 (0.0146)^¶^ | 0.0646 (0.0121)^¶^ | 0.0495 (0.0163)^¶^ | 0.0579 (0.0134)^¶^ |  |  |
| Slight problems to moderate problems |  | 0.1503 (0.0180)^¶^ | 0.1521 (0.0142)^¶^ | 0.1421 (0.0202)^¶^ | 0.1480 (0.0157)^¶^ |  |  |
| Moderate problems to severe problems |  | 0.1285 (0.0172)^¶^ | 0.1273 (0.0135)^¶^ | 0.1445 (0.0193)^¶^ | 0.1406 (0.0150)^¶^ |  |  |
| Severe problems to extreme problems |  | 0.0586 (0.0160)^¶^ | 0.0485 (0.0126)^¶^ | 0.0885 (0.0181)^¶^ | 0.0790 (0.0140)^¶^ |  |  |
| Range of possible values |  | [-0.7872, 1] | [-0.7771, 1] | [-0.9664, 1] | [-0.9583, 1] |  |  |
| Log likelihood |  | -6611.3216 | -4806.9982 | -8061.3658 | -6180.705 |  |  |
| AIC |  | 13262.643 | 9657.9963 | 16164.732 | 12405.41 |  |  |
| BIC |  | 13406.85 | 9816.6238 | 16316.149 | 12564.038 |  |  |

Model estimates are presented as coefficient (SE).

^¶^*p* value <0.01.

AIC, Akaike information criteria; BIC, Bayesian information criteria; GLS, generalized least squares; MAE, mean absolute error; OLS, ordinary least squares; RMSE, root mean square error.

**Table B: Estimation results for DCE (conditional logistic) model (Coefficient as the disutility values)**

|  | DCE modeling (without rescaling) | DCE modeling rescaled using theta derived from hybrid models* |
| --- | --- | --- |
| Mobility (MO) |  |  |
| No problems to slight problem | 0.3460 (0.0702) ^¶^ | 0.0796 (0.0162) ^¶^ |
| Slight problems to moderate problems | 0.4082 (0.0737) ^¶^ | 0.0939 (0.017) ^¶^ |
| Moderate problems to severe problems | 0.7086 (0.0686) ^¶^ | 0.1631 (0.0158) ^¶^ |
| Severe problems to extreme problems | 0.6592 (0.0732) ^¶^ | 0.1517 (0.0168) ^¶^ |
| Self-care (SC) |  |  |
| No problems to slight problem | 0.0483 (0.0764) | 0.0111 (0.0176) |
| Slight problems to moderate problems | 0.2419 (0.0759) ^¶^ | 0.0557 (0.0175) ^¶^ |
| Moderate problems to severe problems | 0.6970 (0.0758) ^¶^ | 0.1604 (0.0174) ^¶^ |
| Severe problems to extreme problems | 0.3254 (0.0702) ^¶^ | 0.0749 (0.0162) ^¶^ |
| Usual Activities(UA) |  |  |
| No problems to slight problem | 0.2123 (0.0698) ^¶^ | 0.0489 (0.0161) ^¶^ |
| Slight problems to moderate problems | 0.0507 (0.0709) | 0.0117 (0.0163) |
| Moderate problems to severe problems | 0.7795 (0.0719) ^¶^ | 0.1794 (0.0165) ^¶^ |
| Severe problems to extreme problems | 0.4366 (0.0738) ^¶^ | 0.1005 (0.0170) ^¶^ |
| Pain/Discomfort (PD) |  |  |
| No problems to slight problem | 0.3320 (0.0725) ^¶^ | 0.0764 (0.0167) ^¶^ |
| Slight problems to moderate problems | 0.1485 (0.0721) | 0.0342 (0.0166) |
| Moderate problems to severe problems | 0.8853 (0.0725) ^¶^ | 0.2038 (0.0167) ^¶^ |
| Severe problems to extreme problems | 0.7303 (0.0762) ^¶^ | 0.1681 (0.0175) ^¶^ |
| Anxiety/depression (AD) |  |  |
| No problems to slight problem | 0.1401 (0.0753) | 0.0322 (0.0173) |
| Slight problems to moderate problems | 0.4965 (0.0721) ^¶^ | 0.1143 (0.0166) ^¶^ |
| Moderate problems to severe problems | 0.6313 (0.0766) ^¶^ | 0.1453 (0.0176) ^¶^ |
| Severe problems to extreme problems | 0.4400 (0.0739) ^¶^ | 0.1013 (0.0170) ^¶^ |
| Range of possible values | [0.0004, 1]^§^ | [-1.0065, 1] |
| Log likelihood | -3076.2541 | -3076.2541 |
| AIC | 6192.5083 | 6192.5083 |
| BIC | 6328.5166 | 6328.5166 |

Model estimates are presented as coefficient (SE).

AIC, Akaike information criteria; BIC, Bayesian information criteria;

^¶^*p* value <0.01.

^§^The possible values represent the probability of choosing one health state over the other.

* In(theta) that we have used for rescaling was derived from hybrid-Tobit het model censored at -1.

**Table C: the raw finding of DCE (conditional logistic) model with intercept constant but without rescaling**


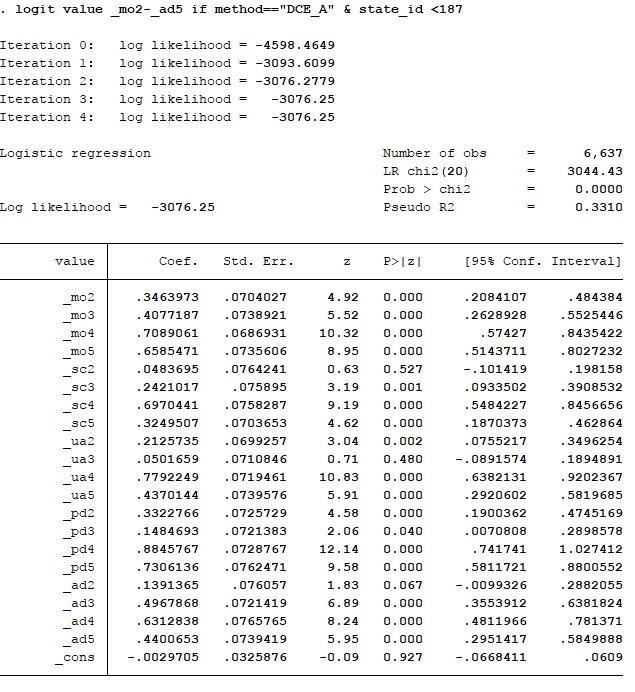


**Table D: Estimation results for hybrid models**

|  | Standard hybrid model | Hybrid model with C-TTO values censored at -1^1^ | Hybrid model with C-TTO values censored at -1 and heteroscedasticity ^2^ |
| --- | --- | --- | --- |
| Mobility (MO) |  |  |  |
| No problems to slight problem | 0.1149 (0.0103) ^¶^ | 0.1076 (0.0112) ^¶^ | 0.0751 (0.0079) ^¶^ |
| Slight problems to moderate problems | 0.0876 (0.0114) ^¶^ | 0.0920 (0.0124) ^¶^ | 0.1046 (0.0126) ^¶^ |
| Moderate problems to severe problems | 0.1565 (0.0114) ^¶^ | 0.1656 (0.0125) ^¶^ | 0.1832 (0.0141) ^¶^ |
| Severe problems to extreme problems | 0.0835 (0.0111) ^¶^ | 0.1115 (0.0122) ^¶^ | 0.1456 (0.0138) ^¶^ |
| Self-care (SC) |  |  |  |
| No problems to slight problem | 0.0801 (0.0100) ^¶^ | 0.0757 (0.0110) ^¶^ | 0.0685 (0.0077) ^¶^ |
| Slight problems to moderate problems | 0.0555 (0.0116) ^¶^ | 0.0565 (0.0127) ^¶^ | 0.0683 (0.0120) ^¶^ |
| Moderate problems to severe problems | 0.1248 (0.0118) ^¶^ | 0.1322 (0.0129) ^¶^ | 0.1463 (0.0138) ^¶^ |
| Severe problems to extreme problems | 0.0325 (0.0107) ^¶^ | 0.0597 (0.0117) ^¶^ | 0.0864 (0.0132) ^¶^ |
| Usual Activities(UA) |  |  |  |
| No problems to slight problem | 0.0780 (0.0102) ^¶^ | 0.0726 (0.0111) ^¶^ | 0.0597 (0.0071) ^¶^ |
| Slight problems to moderate problems | 0.0486 (0.0109) ^¶^ | 0.0508 (0.0119) ^¶^ | 0.0665 (0.0116) ^¶^ |
| Moderate problems to severe problems | 0.1459 (0.0114) ^¶^ | 0.1568 (0.0124) ^¶^ | 0.1777 (0.0135) ^¶^ |
| Severe problems to extreme problems | 0.0478 (0.0113) ^¶^ | 0.0703 (0.0124) ^¶^ | 0.0897 (0.0136) ^¶^ |
| Pain/Discomfort (PD) |  |  |  |
| No problems to slight problem | 0.0899 (0.0099) ^¶^ | 0.0868 (0.0108) ^¶^ | 0.0686 (0.0071) ^¶^ |
| Slight problems to moderate problems | 0.0697 (0.0114) ^¶^ | 0.0710 (0.0124) ^¶^ | 0.0817 (0.0123) ^¶^ |
| Moderate problems to severe problems | 0.1645 (0.0114) ^¶^ | 0.1824 (0.0124) ^¶^ | 0.2011 (0.0137) ^¶^ |
| Severe problems to extreme problems | 0.0887 (0.0116) ^¶^ | 0.1132 (0.0127) ^¶^ | 0.1516 (0.0143) ^¶^ |
| Anxiety/depression (AD) |  |  |  |
| No problems to slight problem | 0.0692 (0.0103) ^¶^ | 0.0637 (0.0113) ^¶^ | 0.0482 (0.0067) ^¶^ |
| Slight problems to moderate problems | 0.1190 (0.0115) ^¶^ | 0.1192 (0.0125) ^¶^ | 0.1400 (0.0119) ^¶^ |
| Moderate problems to severe problems | 0.1444 (0.0113) ^¶^ | 0.1572 (0.0124) ^¶^ | 0.1625 (0.0134) ^¶^ |
| Severe problems to extreme problems | 0.0591 (0.0108) ^¶^ | 0.0811 (0.0119) ^¶^ | 0.1109 (0.0130) ^¶^ |
| Range of possible values | [-0.8602, 1] | [-1.0259, 1] | [-1.2362, 1] |
| Log likelihood | -9797.1085 | -11197.917 | -9089.3232 |
| AIC | 19638.217 | 22439.834 | 20119.296 |
| BIC | 19808.043 | 22609.66 | 20443.51 |

Model estimates are presented as coefficient (SE).

^¶^p value <0.01.

^1^Tobit model (censored at -1) on C-TTO values and same as before for DCE.

^2^Heteroskedastic Tobit model with C-TTO values censored at -1 and same as before for DCE.

AIC, Akaike information criteria; BIC, Bayesian information criteria
